# Successful transvenous pacemaker implantation using a snare-assisted technique in a patient with severe tricuspid stenosis due to bioprosthetic valve dysfunction

**DOI:** 10.1016/j.hrcr.2025.10.001

**Published:** 2025-10-08

**Authors:** Takeshi Mori, Akimitsu Tanaka, Miyuki Ando, Hiroko Goto, Shinijro Miyata, Kazuo Kato

**Affiliations:** Department of Cardiology, Nagoya Tokushukai General Hospital, Kasugai, Aichi, Japan

**Keywords:** Severe tricuspid stenosis, Transvenous pacemaker, Implantation, Bioprosthetic valve dysfunction, Tricuspid valve replacement, Snare-assisted lead placement

**Key Teaching Points**

• We report a case of complete atrioventricular (AV) block in a patient with severe tricuspid stenosis caused by bioprosthetic valve dysfunction after tricuspid valve replacement. A transvenous pacemaker was implanted to treat the AV block.
• Advancing the lead through the valve was challenging owing to a severely dilated right atrium and stenotic tricuspid valve. Therefore, we used the TSURECOMI technique to successfully position the right ventricular (RV) lead in the RV.
• Although leadless pacemakers and coronary sinus lead placement are alternative options, successful implantation is not always guaranteed, particularly in anatomically complex cases. We believe that the TSURECOMI technique, as demonstrated here, provides a valuable bailout strategy for RV lead placement when other approaches are not feasible.

## Introduction

Severe tricuspid stenosis is relatively rare, with limited evidence regarding optimal treatment strategies. Although rheumatic disease is the most common cause of acquired tricuspid stenosis,^1^ it can also result from bioprosthetic valve dysfunction after tricuspid valve replacement (TVR). Surgical intervention on the tricuspid valve (TV) carries a high risk of atrioventricular block, often requiring permanent pacemaker implantation.^2^ However, to the best of our knowledge, no previous reports have described transvenous pacemaker implantation in patients with severe tricuspid stenosis caused by bioprosthetic valve dysfunction. This may be partly because advancing a pacing lead through a markedly dilated right atrium and stenotic TV is technically difficult. The TSURECOMI technique was originally developed in the context of transcatheter aortic valve implantation (TAVI) as a bailout strategy, where a snare is used to reintroduce a guidewire into the left ventricle (LV) after it has slipped back into the aorta.^3^ This technique enables controlled advancement of a device or lead from a dilated chamber through a narrow or stenotic segment, making it useful in anatomically complex situations. Herein, we report a case of successful transvenous pacemaker implantation using the TSURECOMI technique in a patient with severe tricuspid stenosis.

## Case report

**Figure 1:**
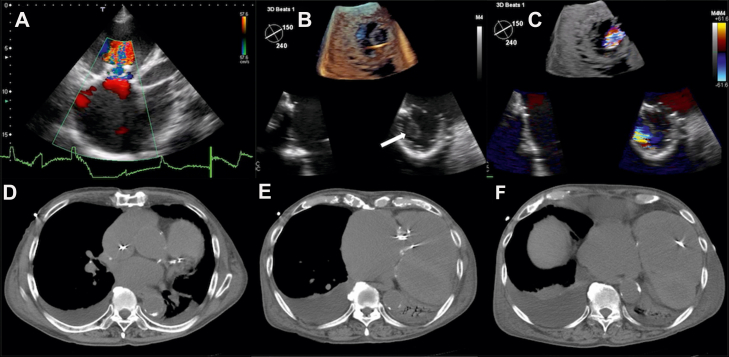
Multimodal imaging demonstrating tricuspid stenosis and right atrial enlargement. Transthoracic echocardiography (**A**) demonstrates accelerated flow across the tricuspid valve into the right ventricle. Transesophageal echocardiography (**B, C**) shows incomplete opening of the bioprosthetic valve, with only 1 leaflet visualized as open (*white arrow* in panel B). Chest computed tomography images after temporary pacemaker insertion (**D–F**) reveal a markedly enlarged right atrium.

An 83-year-old man presented to our hospital with dyspnea that had persisted for 3 days. His medical history included surgical TVR with a bioprosthetic valve 10 years earlier for severe tricuspid regurgitation. The patient was prescribed carvedilol 5.0 mg/d and furosemide 20 mg/d at a local clinic and was followed up with an annual visit to our hospital’s cardiac surgery outpatient clinic, without problems. Chest radiography revealed pulmonary congestion and bilateral pleural effusion, and chest auscultation revealed coarse crackles in both lung fields. His heart rate was 36 beats/min, and electrocardiography showed complete atrioventricular block. Blood tests revealed severe renal dysfunction (blood urea nitrogen, 72.4 mg/dL; creatinine, 2.39 mg/dL; and estimated glomerular filtration rate, 21). Transthoracic echocardiography demonstrated marked right atrial enlargement and moderate tricuspid regurgitation, with a tricuspid regurgitant pressure gradient of 50 mm Hg (Figure 1A). Chest computed tomography revealed significant right atrial and right ventricular (RV) enlargement (Figure 1D–1F). To evaluate pulmonary hypertension, right heart catheterization was performed on the day of admission, and a temporary pacemaker was implanted for complete atrioventricular block. Both procedures were technically difficult owing to marked right atrial enlargement, which caused catheter entrapment within the atrial chamber; however, both were eventually completed successfully. Suspecting bioprosthetic valve dysfunction, transesophageal echocardiography was performed on the day after admission, which revealed severe tricuspid stenosis (TV area, 0.66 cm^2^; peak velocity, 1.4 m/s; and mean pressure gradient, 5 mm Hg) (Figure 1B and 1C). It was later determined that the procedural challenges during right heart catheterization and temporary pacemaker implantation were not only caused by atrial enlargement but also by significant tricuspid stenosis. Although TV re-replacement was considered, the patient had decompensated heart failure and was considered a high surgical risk owing to advanced age and the need for redo sternotomy. After confirming the patient and family’s wishes, the heart team, comprising cardiovascular surgery, anesthesiology, and cardiology departments, decided on permanent pacemaker implantation as the first-line treatment to stabilize heart failure, with valve re-replacement considered if heart failure proved refractory.

On the 14th day of hospitalization, after preoperative antibiotic administration, mild sedation was achieved with midazolam and dexmedetomidine, followed by a local anesthetic incision in the left anterior subclavicular area. Owing to severe renal dysfunction, venous tracking with contrast was avoided. A 4F pigtail catheter was advanced from the right common femoral vein to the left subclavian vein and used as a landmark for venous access via the Seldinger technique. In this case, Solia S60 (Biotronik, Berlin, Germany) was used. Owing to TV narrowing and right atrial enlargement, advancing the lead across the valve with a stylet alone was difficult.

**Figure 2:**
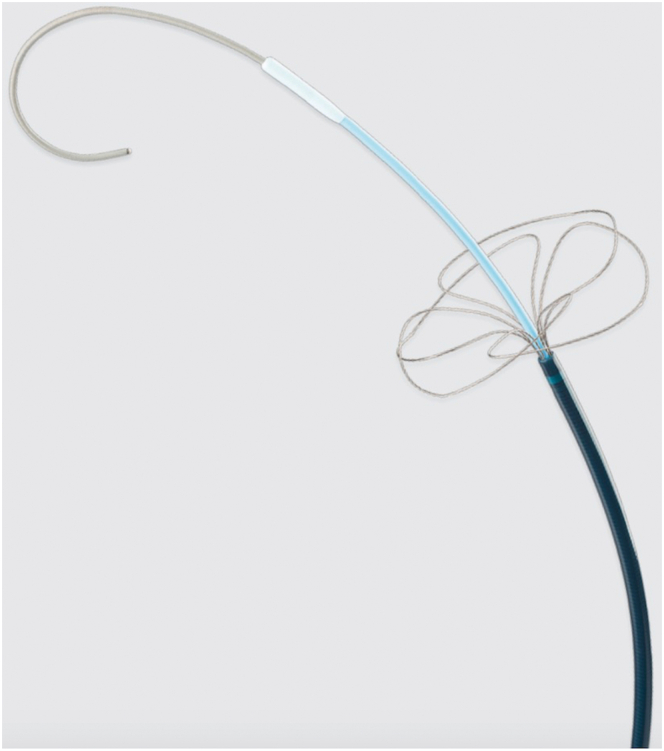
INDY OTW vascular retriever used in the snare-assisted technique. The INDY OTW vascular retriever is an over-the-wire snare, used in the TSURECOMI technique.

**Figure 3:**
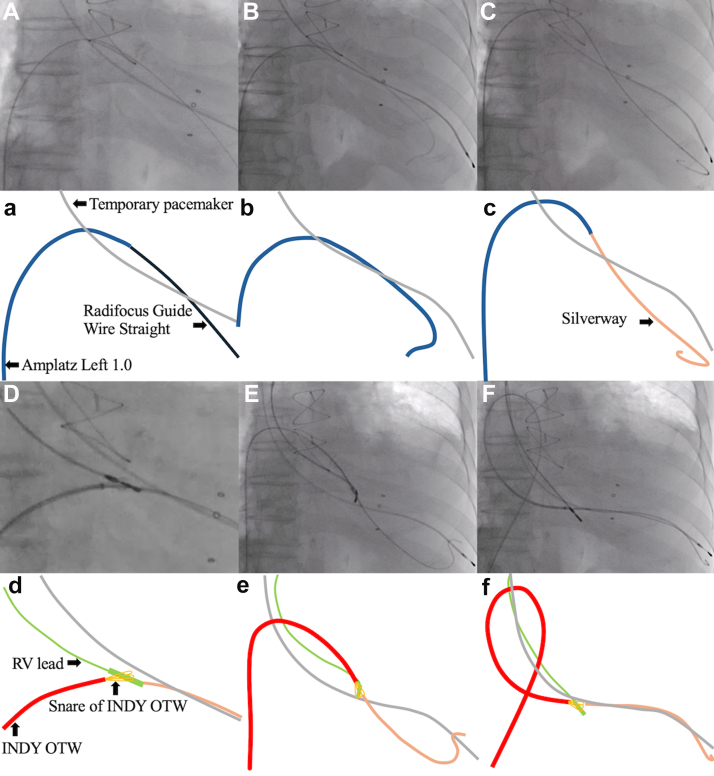
Initial attempt to advance the RV lead across the stenotic tricuspid valve. Panels A–E are fluoroscopic images, whereas panels a–e show simplified schematics. Using a 4F Amplatz Left (AL) 1.0 catheter and a Radifocus Guide Wire Straight, we successfully crossed the stenosed prosthetic valve (**A**). The AL catheter was advanced to the RV (**B**), and the guide wire was subsequently exchanged for a Silverway (**C**). The lead was snared in the right atrium using an INDY OTW catheter (**D**). Advancing the lead into the RV was challenging (**E and F**). Panels E and F show sequential images of this maneuver. RV = right ventricle.

**Figure 4:**
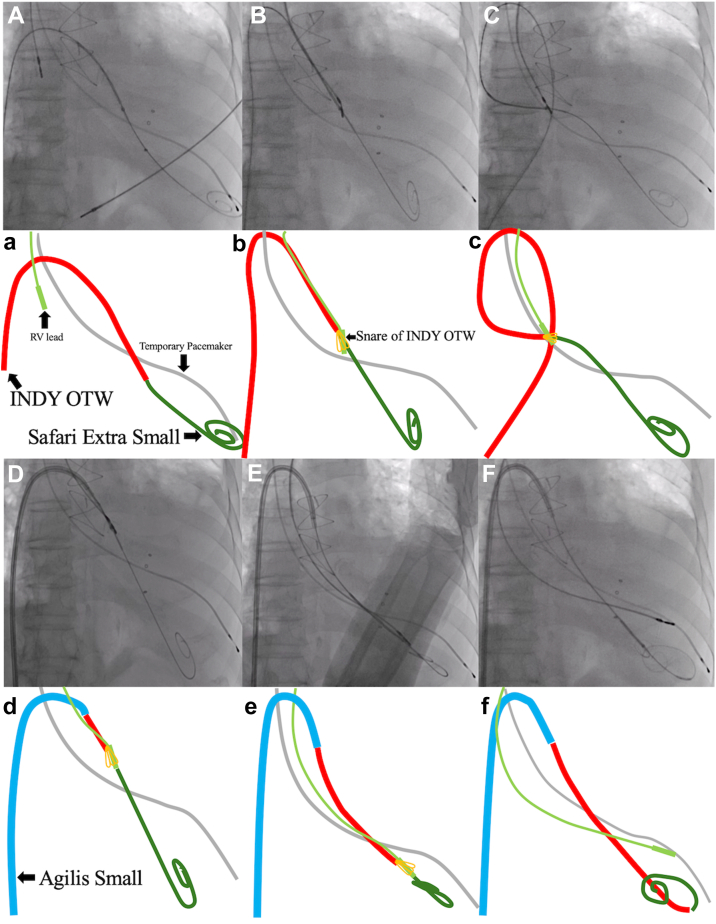
Use of steerable sheath and snare to facilitate lead advancement. Panels A–E are fluoroscopic images, whereas panels a–e show simplified schematics. The Silverway wire was exchanged for a Safari XS using the INDY OTW catheter (**A**). Despite this, attempts to advance the lead into the RV, while it remained snared in the right atrium, were unsuccessful (**B and C**). A steerable Agilis Small sheath was then inserted (**D**), which provided additional support. With simultaneous forward pressure applied to the RV lead and traction from the snare, the lead was successfully advanced into the RV (**E**). The lead was carefully unsnared within the RV (**F**). RV = right ventricle.

**Figure 5:**
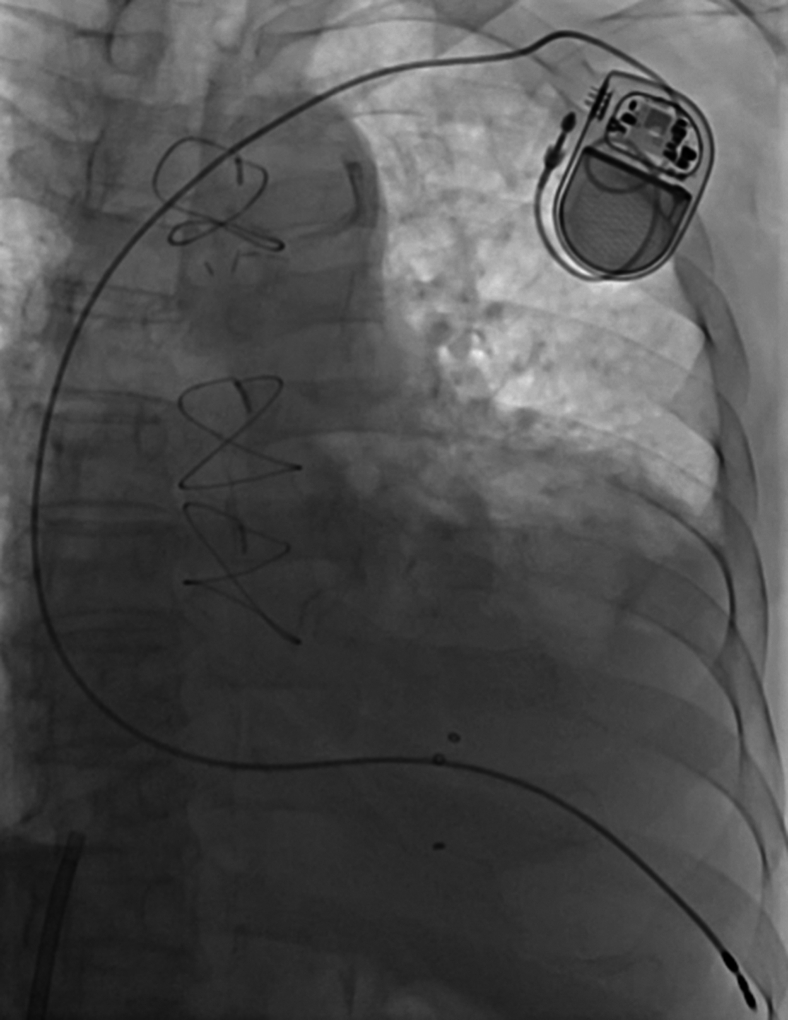
Final lead position at the right ventricular apex. Implantation of the lead into the right ventricular septum proved difficult. Therefore, the lead was ultimately positioned at the right ventricular apex. All electrical parameters, including pacing threshold, R-wave amplitude, and lead impedance, were within acceptable limits.

Therefore, we decided to advance the lead to the RV using the TSURECOMI technique.^3^ The “TSURECOMI” technique (a transarterial snare-upholding recovery technique for completely pulled out LV wire for transcatheter aortic valve replacement valve insert system) was originally developed in the context of TAVI. This method involves using an INDY OTW (Cook Medical, Indiana) to grasp the TAVI delivery system and using it as a “rail” to readvance the valve or a new guidewire into the LV (Figure 2). An 8F sheath was first introduced into the right common femoral vein. A 4F Amplatz Left 1.0 catheter and a Radifocus Guide Wire Straight (Terumo, Japan) were used to cross the TV (Figure 3A). The wire was then exchanged for a Silverway (ASAHI INTEC, Japan), and an INDY OTW was used to grasp the RV lead and pull it across the stenotic TV (Figure 3). Despite this, advancing the lead remained challenging. To address this, the guidewire was exchanged for a Safari Extra Small (Boston Scientific, Massachusetts), a stiffer wire, and the sheath was replaced with an Agilis Small steerable sheath (Abbott, St. Paul, MN) (Figure 4). Therefore, we successfully advanced the RV lead by pushing the INDY OTW (which was holding the lead) and the lead simultaneously. The lead was positioned at the RV apex where we could obtain the appropriate pacing threshold, R-wave amplitude, and impedance values. The INDY OTW was then exchanged for a 4F Amplatz Left 1.0 catheter to carefully retrieve the Safari wire, avoiding entanglement with the RV lead. Pacemaker implantation was successfully completed in 2.5 hours (Figure 5).

The patient received guideline-directed therapy for heart failure since admission. After pacemaker implantation, rehabilitation was expanded, and shortness of breath improved. He was discharged on postoperative day 20 and monitored as an outpatient. No heart failure occurred, but renal function gradually deteriorated. Maintenance hemodialysis was suggested but was not desired. The patient died of multiple organ failure 7 months after discharge.

## Discussion

Patients undergoing surgical intervention for the TV are at an elevated risk of atrioventricular conduction disturbances, with reported incidences of 24%–26% after TVR.^4^^,^^5^ However, no reliable patient-specific predictors of postoperative atrioventricular conduction block currently exist.^6^ Consequently, there is no established consensus on whether epicardial leads should be implanted during surgery or whether transvenous pacemakers should be inserted postoperatively.^7^ This case report describes a complete atrioventricular block occurring in the remote phase after TVR, associated with tricuspid stenosis caused by bioprosthetic valve dysfunction. Although TVR was viable, the patient’s advanced age and poor general condition and the family’s request led to avoiding the high risks of repeat sternotomy. Instead, pacemaker implantation was pursued for heart failure treatment. A previous report described transvenous lead pacemaker implantation for complete atrioventricular block after transcatheter edge-to-edge repair for TV regurgitation,^8^ in which a 3-dimensional (3D)-shaped stylet was used to cross the TV. In this case, it was difficult to pass the lead using the stylet alone.

Several companies offer specialized sheaths for septal lead placement. We considered using a guidewire to cross the TV with a septal sheath and then position the lead. However, the markedly enlarged right atrium raised concerns about lead prolapse into the inferior vena cava, whereas the severely narrowed valve orifice presented a significant risk of sheath entrapment. The only 3D preshaped sheath available for use was 39 cm, and with the enlarged right atrium, its length might have been insufficient. Selecting a specific local site, such as the left bundle area, for lead placement was also difficult. The patient had moderate tricuspid regurgitation, and advancing a short sheath through the stenotic TV to the RV outflow could risk bioprosthetic valve damage and worsening regurgitation. Therefore, a septal sheath was not used.

A previous report described LV lead insertion from the coronary sinus (CS) using intracardiac echocardiography and 3D mapping for bioprosthetic TV dysfunction.^9^ In this case, an alternative approach to the LV lead in the CS was considered. However, contrast imaging is required to confirm lead position even in accessible veins (eg, anterior interventricular or middle cardiac vein). Because the patient’s renal function was severely impaired, contrast use risked further deterioration of kidney function; thus, this approach was avoided. In addition, there was the possibility that even if the CS was accessed using an LV lead, an adequate threshold might not be achieved, potentially necessitating a new RV lead to achieve pacing. From an economic standpoint, the placement of an LV lead in the CS was deemed unreasonable; therefore, it was not performed in this case.

Although leadless pacemaker implantation has been reported in a patient with tricuspid stenosis,^10^ this approach was not selected. A massively enlarged right atrium and stenotic TV would complicate transvalvular delivery, prolonging procedural time and increasing sheath-related risks such as impaction or valve injury. If the leadless pacemaker can only be implanted close to the TV, interfering with it, an object thicker than a transvenous lead would occupy the already narrowed TV, increasing the possibility of further stenosis. In addition, if the leadless pacemaker is completely unsuccessful, it would require a new RV lead or generator, which would be likewise economically unreasonable. Despite these concerns, the possibility of worsening tricuspid stenosis from a transvenous lead was also considered and explained to the patient’s family. Serial transthoracic echocardiography after temporary 5F pacing lead placement showed no progression in the degree of tricuspid stenosis. Given that the permanent RV lead used was 5.6F, with minimal size difference, significant progression of stenosis owing to the RV lead was not a major concern. Ultimately, a transvenous lead system was chosen as the more reliable, less invasive option, given the anatomic challenges and risks associated with both strategies in this patient population.

The TSURECOMI technique, originally developed for TAVI, involves pulling and guiding a target object into position using a snare. In cases requiring navigation through a markedly enlarged right atrium and stenotic TV, the TSURECOMI technique can be particularly effective. Although leadless pacemakers are gaining prominence, deployment may fail with significant right atrial enlargement. In such situations, a bailout strategy like the one used here may serve as a valuable alternative.

One limitation of this case report is that transesophageal echocardiography was not performed after pacemaker lead placement in the chronic phase, making it difficult to determine the appropriateness of a transvenous lead for long-term prognosis. Although tricuspid stenosis associated with bioprosthetic valve dysfunction typically requires surgery, the patient’s overall condition necessitated a nonsurgical approach in this case. Given that tricuspid stenosis is uncommon, comparing long-term prognosis with that of other alternatives, such as LV lead or leadless pacemaker implantation, is difficult. However, no remarkable changes were observed on transthoracic echocardiography, and there was no worsening of right heart failure symptoms, such as lower limb or facial edema. In fact, the patient’s shortness of breath tended to improve after pacemaker implantation. Thus, we consider that the outcomes were acceptable in the midterm phase.

## Conclusion

In this case of severe tricuspid stenosis caused by bioprosthetic valve dysfunction, the TSURECOMI technique enabled successful placement of a transvenous lead. We propose that this method is a viable option for pacemaker implantation when using leadless systems is not feasible in patients with severe tricuspid stenosis.

## Disclosures

The authors have no conflicts of interest to disclose.
